# Obesity drives dysregulation in DC responses to viral infection

**DOI:** 10.1093/discim/kyaf001

**Published:** 2025-02-06

**Authors:** Andrea Woodcock, Ronan Bergin, Nidhi Kedia-Mehta, Cathriona Foley, John C Stephens, Donal O’Shea, Mary Canavan, Andrew E Hogan

**Affiliations:** Kathleen Lonsdale Institute for Human Health Research, Maynooth University, Maynooth, County Kildare, Ireland; Translational Immunopathology, School of Biochemistry & Immunology, Trinity Biomedical Sciences Institute, Trinity College Dublin, Dublin, Ireland; Kathleen Lonsdale Institute for Human Health Research, Maynooth University, Maynooth, County Kildare, Ireland; Kathleen Lonsdale Institute for Human Health Research, Maynooth University, Maynooth, County Kildare, Ireland; Department of Surgery, School of Medicine, University College Cork, Cork, Ireland; Kathleen Lonsdale Institute for Human Health Research, Maynooth University, Maynooth, County Kildare, Ireland; Kathleen Lonsdale Institute for Human Health Research, Maynooth University, Maynooth, County Kildare, Ireland; St. Vincent’s University Hospital and University College Dublin, Dublin, Ireland; Translational Immunopathology, School of Biochemistry & Immunology, Trinity Biomedical Sciences Institute, Trinity College Dublin, Dublin, Ireland; Kathleen Lonsdale Institute for Human Health Research, Maynooth University, Maynooth, County Kildare, Ireland; St. Vincent’s University Hospital and University College Dublin, Dublin, Ireland

**Keywords:** dendritic cells, immunometabolism, metabolism, Sendai virus

## Abstract

**Introduction:**

Obesity is a worldwide epidemic, with over 1 billion people worldwide living with obesity. It is associated with an increased risk of over 200 chronic co-morbidities, including an increased susceptibility to infection. Numerous studies have highlighted the dysfunction caused by obesity on a wide range of immune cell subsets, including dendritic cells (DCs). DCs are innate immune sentinels that bridge the innate and adaptive immune systems. DCs provide critical signals that instruct and shape the immune response. Our group has previously reported that DCs from people with obesity display defective cytokine production; however, the mechanisms underpinning these defects are unclear.

**Methods:**

We investigated the functional responses of DCs using a murine-specific single-stranded RNA virus, Sendai virus, in mice on a standard diet and in a model of diet-induced obesity.

**Results:**

Here, we demonstrate that GM-CSF cultured bone marrow–derived DCs (GM-DCs) from mice on a high-fat diet (HFD) have reduced cytokine production following viral challenge. This was associated with a dysfunctional metabolism through reduced translation in the HFD GM-DCs.

**Conclusions:**

We propose that obesity-mediated effects on DCs have downstream consequences on their ability to effectively mediate subsequent immune responses, especially during viral infection.

## Graphical Abstract

**Graphical Abstract:**
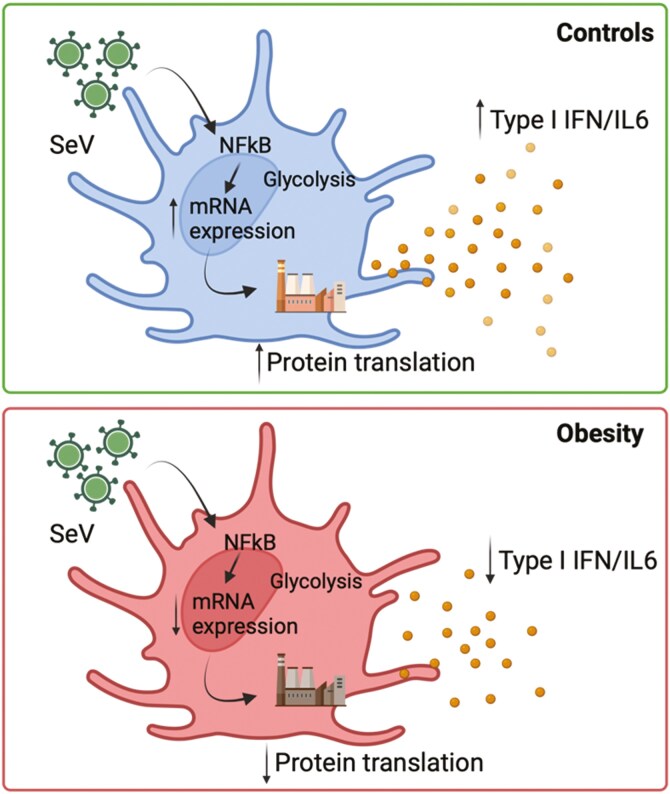

## Introduction

Obesity is a multi-factorial, chronic, and progressive disease, which currently affects over 1 billion people worldwide [1]. A defining feature of obesity is a chronic, low-grade inflammation which is typically characterized by increased pro-inflammatory cytokine secretion and the infiltration of leukocytes, into the adipose tissue [2]. Furthermore, we know that there is a clear association between obesity and various non-communicable diseases such as type 2 diabetes mellitus, cardiovascular disease, and cancer [3, 4]. Importantly, people with obesity (PWO) have an increased susceptibility to infection, resulting in increased morbidity and mortality, as demonstrated during the H1N1 infection and coronavirus disease 2019 (COVID-19) pandemics [5, 6].

It is well established that obesity-driven immune dysregulation plays a major role in the development of these co-morbidities. Obesity has a myriad of effects on immune cells, mainly deleterious [7–9]. Obesity-associated lipid accumulation within natural killer cells impedes their function, demonstrated via reduced interferon (IFN)-γ production [10]. Obesity is also associated with macrophage polarization towards a more inflammatory phenotype [7]. The inability of immune cells to mount adequate or appropriate immune responses against pathogens in PWO is understandably a significant cause for concern. Alongside the increased susceptibility to infection, PWO also display reduced antibody titres following vaccination [11–13]. A year after vaccination, individuals with an increased body mass index demonstrated a significant decrease in influenza-specific antibody titres [11]. Studies in mice with diet-induced obesity (DIO) additionally demonstrated altered T-cell-mediated immune responses [14, 15], where mice with DIO displayed reduced influenza-specific memory T-cell function [14].

DCs are professional antigen-presenting cells with the particular ability of inducing the differentiation of naïve T cells into memory T cells [16], a crucial component of successful vaccination strategies [17]. Immature DCs act as sentinels, circulating the body and entering tissues where pathogen encounter is most likely [16, 18]. In an immature or resting state, DCs are more phagocytic and have reduced stimulatory properties due to low expression of antigen presentation and co-stimulatory molecules [16], allowing antigen detection. Upon antigen uptake and processing, the DCs are activated and undergo maturation where the cells upregulate their antigen presentation machinery, including MHC molecules, co-stimulatory molecules, and cytokines [16].

Previous human and animal studies have explored the impact of obesity on DCs and have detailed defects that are context- and location-dependent. A decrease in DCs was observed in the circulation of PWO, along with a reduction in the expression of maturation marker, CD83, following TLR stimulation [19]. However, increased adipose tissue DCs were observed in both humans and mice [20]. The molecular mechanism underpinning these alterations in DCs in the setting of obesity remains unclear. DC maturation involves an upregulation of mRNA transcripts and vast protein production; therefore, the cells must drastically increase their metabolism, to meet the energetic and metabolic demands of the cell. As obesity has been found to affect the metabolism of various immune cells [10, 21, 22], we hypothesized that obesity could impact DC metabolism, underpinning in part the defects reported in DCs.

To explore this further, we investigated the impact of obesity on murine GM-CSF cultured bone marrow–derived DCs (GM-DCs) and their subsequent challenge *in vitro* with Sendai virus (SeV), a negative sense single-stranded RNA virus (ssRNA) [23]. GM-DCs resemble *in vivo* monocyte-derived DCs (Mo-DCs), Mo-DCs are a group of cells that were found to differentiate during infection from monocytes or during inflammation [24]. Mo-DCs have a conventional DC (cDC)-like and inflammatory phenotype. SeV is a murine-specific, potent activator of DCs [25] and a strong inducer of IFN-β [26]. SeV is useful to study the effects of ssRNA viruses, as there are many disease-causing RNA viruses, for example, influenza or HIV [27]. We found that the SeV challenge elicits a robust antiviral response in GM-DCs with increased production of key cytokines such as TNF and IFN-β. We demonstrate that this antiviral response is dependent on metabolic rewiring, in particular glucose metabolism. Finally, we demonstrate that obesity limits the antiviral response, and this is paired with diminished protein translation.

## Methods and materials

### Mice and diet-induced model of obesity

Male C57BL6/J mice were purchased from Charles River Laboratories at 6–8 weeks of age. Mice were acclimatized for 1 week prior to the initiation of experiments. Following acclimatization, mice were fed either the standard diet (SD; 18% kcal of fat, T2918, Envigo) or the high-fat diet (HFD; 60% kcal of fat, TD.06414, Envigo) ad libitum. Mice were fed their respective diets for 16 weeks. Five representative mice were weighed from each group each week from Week 0 (start of diet). Mice were culled via cervical dislocation.

### Generation of GM-DCs

Mice were sacrificed and the femur and tibia of the mice were flushed with sterile PBS using a 27¾ gauge needle to isolate the bone marrow precursors. Cells were washed and plated in complete medium (RPMI-1640 plus GlutaMAX [Thermo Fisher Scientific]), supplemented with penicillin (100 I.U./ml) and streptomycin (100 μg/ml, Sigma-Aldrich), 10% v/v premium foetal bovine serum (Thermo Fisher Scientific) and recombinant mouse GM-CSF (20 ng/ml, BioLegend). Fresh complete medium was added on Day 3 and Day 7. On Day 6, supernatants were discarded, and the complete culture medium was entirely replaced. On Day 10, culture supernatant containing non-adherent and loosely adherent cells was harvested, washed, and resuspended in fresh medium with GM-CSF (10 ng/ml) for subsequent experiments. Sendai virus Cantell strain was gifted from Professor Paul Moynagh (Maynooth University, Co. Kildare, Ireland). SeV is also known as murine respirovirus. SeV was obtained at a hemagglutination (HA) tube titre of 4000/ml.

### Enzyme-Linked Immunosorbent Assay

GM-DCs were seeded (1 × 10^6^ cells/ml; 200 μl/well) in 96-well plates and allowed to rest for 1–2 hours. Cells were infected with SeV at a 1:1000 dilution, in the presence or absence of metabolic inhibitors, 2-Deoxy-D-glucose (2-DG; 1 mM, Sigma-Aldrich), oligomycin (1 μM, Sigma-Aldrich), and heptelidic acid (5 μM, Abcam) for 18 hours. All conditions were plated in triplicates. Supernatants were collected and IFN-β, IL-6, and TNF-α were quantified by sandwich ELISA (R&D systems) as per the manufacturer’s instructions.

### RNA

GM-DCs were seeded (1 × 10^6^ cells/ml; 3 ml/well) in 6-well plates, rested, and infected with SeV at 1:1000 dilution, in the absence or presence of 2-DG and oligomycin for 3, 6, and 18 hours. Total RNA was extracted from cells using TRIzol Reagent (ThermoFisher Scientific) and ThermoFIsher Scientific’s protocol. cDNA was generated from 500 ng of RNA using the qScript cDNA Synthesis kit (QuantaBio) and real-time PCR analyses were performed using PerfeCTa SYBR Green FastMix ROX Reaction Mix (QuantaBio) and KiCqStart primer sets (Sigma-Aldrich) and an Applied Biosystems StepOnePlus Real-Time PCR System. Expression levels were normalized relative to the housekeeping gene *actin beta* (*Actb).*

### RNA sequencing

RNA isolated from basal and 18-hour SeV-treated SD and HFD GM-DCs was sequenced by Novogene (UK) Company Limited on an Agilent 5400 Fragment Analyzer system. Cleaned data were aligned to the reference mouse (*Mus musculus*) genome (ensembl_mus_musculus_grcm38_p6_gca_000001635_8) using the alignment program HISAT2. Alignment was proceeded by quantification of gene expression. The gene counts were inputted into edgeR and normalized using the Voom R package. The Log_2_FoldChange, *P*-values, and false discovery rate were calculated. Differential expression analysis was performed between two conditions, each with three biological replicates: SD GM-DCs untreated versus SeV-stimulated; SD GM-DCs untreated versus HFD GM-DCs untreated; and SD GM-DCs SeV-stimulated versus HFD GM-DCs SeV-stimulated. Gene Set Enrichment Analysis (GSEA) was performed on normalized gene counts, and mouse hallmark gene sets from the Molecular Signatures Database (MSigDB) were used to determine differences in genes within specific cellular pathways. Results from RNA sequencing data were displayed as heatmaps or volcano plots of differentially expressed genes. Heatmaps were produced using Morpheus (https://software.broadinstitute.org/morpheus) and volcano plots were produced using Graph Pad Prism 10 software.

### Single-cell energetic metabolism by profiling translation inhibition

GM-DCs were seeded (1 × 10^6^ cells/ml; 2 ml/well), rested, and infected with SeV at 1:1000 dilution for 18 hours. Following stimulation, the Single-Cell ENergetIc metabolism by profilIng Translation inhibition (SCENITH) assay was performed as per Argüello *et al.* [28], including the calculation of the metabolic dependencies and capacities. The cells were divided amongst five wells: control, 2-DG, Oligomycin, 2-DG & Oligomycin, and no puromycin wells. Inhibition concentrations were 100 mM of 2-DG and/or 1 μM of oligomycin. The cells were incubated with their respective inhibitors for 15 minutes at 37°C. Followed by the addition of puromycin (11 μM, Sigma-Aldrich) to all wells, except no puromycin wells, for 25 minutes at 37°C. Ice-cold PBS stopped puromycin incorporation. Subsequently, extracellular staining was performed (anti-CD11c FITC, anti-MHC II VioBlue, anti-CD40 PE, anti-CD86 Pe-Cy7, eFluor506 Viability dye). Following staining, cells were fixed and permeabilized using the True-Nuclear Transcription Factor Buffer set (BioLegend). The cells were stained intracellularly with anti-puromycin AlexaFluor488 (Sigma-Aldrich). Flow cytometry was performed using the Attune Nxt Flow Cytometer, and results analysed using FlowJo software (Treestar).

### Immunoblotting

GM-DCs were seeded (1 × 10^6^ cells/ml; 3 ml/well), rested, and infected with SeV at 1:1000 dilution for 3 and 6 hours. Cells were lysed in 2X Laemmli sample buffer (4X: 0.25M Tris-base (pH 6.8), 6% (w/v) SDS, 40% (w/v) glycerol (Sigma-Aldrich), 0.04% Bromophenol blue (Sigma-Aldrich), 20% 2-mercaptoethanol (Sigma-Aldrich) in dH_2_O, and 2X: one in two dilution of 4X in dH_2_O). Samples were resolved by SDS-PAGE and transferred to nitrocellulose membranes as per Bio-Rad’s instructions. Followed by immunoblot analysis with anti-phospho-IκBα Rabbit (Cell Signaling Technology) and anti-β-Actin Mouse (Sigma-Aldrich) antibodies. Protein bands were visualized by enhanced chemiluminescence. Densitometry analysis was performed using ImageJ software. β-actin was used to normalize the target protein, phospho-IκBα (p-IκBα), levels.

### Statistical analysis

Graph Pad Prism 10 software was used for data visualization and statistical analysis. Data are expressed as mean ± standard error mean. Student’s *t*-test was used for differences between the two groups. Analysis of three or more groups was determined using ANOVA. Statistical significance was denoted as *P* < 0.05.

## Results

### Infection with the murine respiratory virus Sendai (SeV) is a robust inducer of immunomodulatory responses in DC

**Figure 1:**
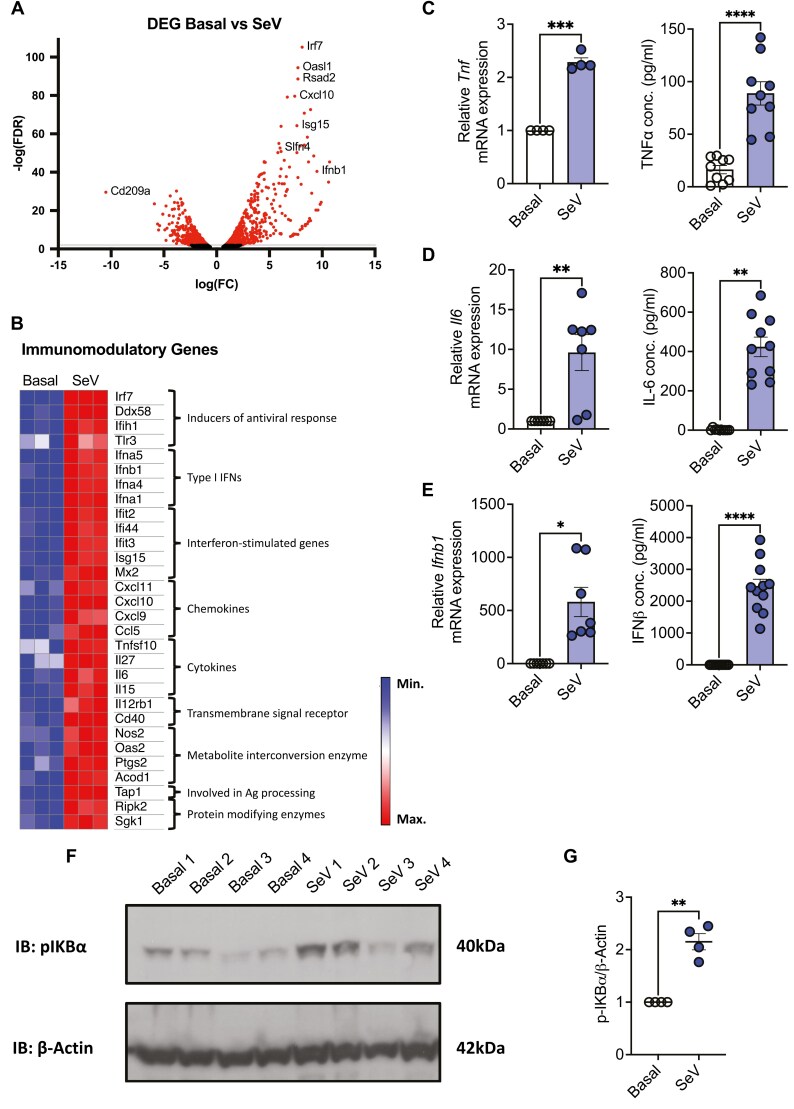
SeV drives immune responses in GM-DCs. (A) Volcano plot depicting differentially expressed genes (DEGs) from SeV-stimulated GM-DCs compared to basal generated from RNA sequencing data. (B) Heat map of key DEGs involved in GM-DC viral activation, gene counts, obtained from RNA sequencing data, are normalized with an FDR < 0.1 and log_2_fold change either ≥1.5 or ≤−1.5 (*n* = 3). (C) RT-PCR of *Tnf* mRNA expression relative to *Actb* following 6-hour SeV stimulation (*n* = 4) and TNFα production (pg/ml) following 18-hour SeV stimulation (*n* = 9). (D) RT-PCR of *Il6* mRNA expression relative to *Actb* following 6-hour SeV stimulation (*n* = 7) and IL-6 production (pg/ml) following 18-hour SeV stimulation (*n* = 10). (E) RT-PCR of *Ifnb1* mRNA expression relative to *Actb* following 6-hour SeV stimulation (*n* = 7) and IFNβ production (pg/ml) following 18-hour SeV stimulation (*n* = 11). (F) Immunoblot of phospho-IκBα (40kDa) and β-Actin (42kDa) expression basally and following 6-hour SeV stimulation. (G) Densitometry of p-IκBα expression relative to β-Actin (*n* = 4). Statistical analysis performed using paired student’s *t*-test or Wilcoxon test where appropriate.

While SeV infection in mice is a known inducer of type I interferons [26], the phenotypic, functional, or indeed transcriptomic effects of infection on DC responses are relatively unexplored. We examined the transcriptional and immunomodulatory effects of SeV infection on GM-DCs. We demonstrate that transcriptional remodelling occurs following SeV stimulation of GM-DCs (Fig. 1A and B). Specifically following 18-hour SeV stimulation, 1293 genes were significantly increased in expression concomitant with a significant decrease in 1528 genes, compared to untreated GM-DCs (Fig. 1A and B and Supplementary Table 1). Additionally, to further examine response-specific differences in GM-DC gene expression following SeV infection at the pathway level between basal and SeV-treated GM-DC, GSEA was performed.

Based on results obtained from GSEA, we examined specific genes associated with these pathways and genes required for DC activation or their antiviral response (Fig. 1B). We identified significant enrichment in genes associated with DC maturation, activation, and cytokine production. Specifically, there was an enrichment in genes involved in antigen processing (*Tap1*), type I interferon production (*Ifna1, Ifna4, Ifna5, Ifnb1*), chemokine (*Cxcl9*, *Cxcl10*, *Cxcl11*), and cytokine production (*Il27, Il6, Il15*) in addition to interferon-stimulated genes (*Isg15*). Specifically, *DEAD Box Protein 58* (*Ddx58)* (also known as Rig-I), *Interferon Induced with Helicase C Domain 1 (Ifih1)* (also known as Mda-5), and *Tlr3* are also induced upon SeV infection and function to detect viral nucleic acids. SeV replication can result in the production of defective interfering RNA (DI RNA) which can activate RIG-I and MDA-5, these viral sensors lead to the activation of transcription factors, NF-κB and IRF3 [29, 30]. These transcription factors activate target genes involved in pro-inflammatory and antiviral responses, such as cytokines (IFNs, TNFα) or chemokines [31]. Collectively the induction of these genes is critical for antiviral immune responses in DCs [32, 33].

To examine if this transcriptional diversity in SeV-infected DC translates into functional diversity, in terms of DC activation, we performed subsequent gene and protein analysis to measure cytokine levels in GM-DCs in response to SeV (Fig. 1C–E). Upon SeV stimulation across varying time points, we demonstrated peak cytokine mRNA expression at 6 hours. We demonstrated a significant increase in pro-inflammatory and antiviral cytokines, *Tnf*, *Il6*, and *Ifnb1* (*P* < 0.05) via RT-PCR, in agreement with our transcriptomic analysis (Fig. 1C–E). Following 18-hour SeV stimulation, the corresponding cytokine protein levels were significantly increased (*P* < 0.05), ensuring the GM-DCs are equipped for subsequent antiviral responses, such as the activation of adaptive immune cells (Fig. 1C–E). To understand the dominant intracellular signalling pathways which may be responsible for this response we investigated the effect of SeV stimulation on the NF-κB pathway. NF-κB is a master immune regulator with a plethora of diverse immunological related genes under its control [31]. Activation of NF-κB involves the phosphorylation and subsequent proteolytic degradation of the inhibitory protein IκB by specific IκB kinases [31]. We measured IκBα phosphorylation and demonstrated that SeV stimulation significantly increased the phosphorylation of IκBα compared to untreated GM-DCs (*P* < 0.01; Fig. 1F and G). Therefore, suggestive that the antiviral response in SeV-stimulated GM-DCs may be mediated in part via the NF-κB pathway.

### Metabolic rewiring through glycolysis is required for SeV-induced cytokine responses in DC

**Figure 2:**
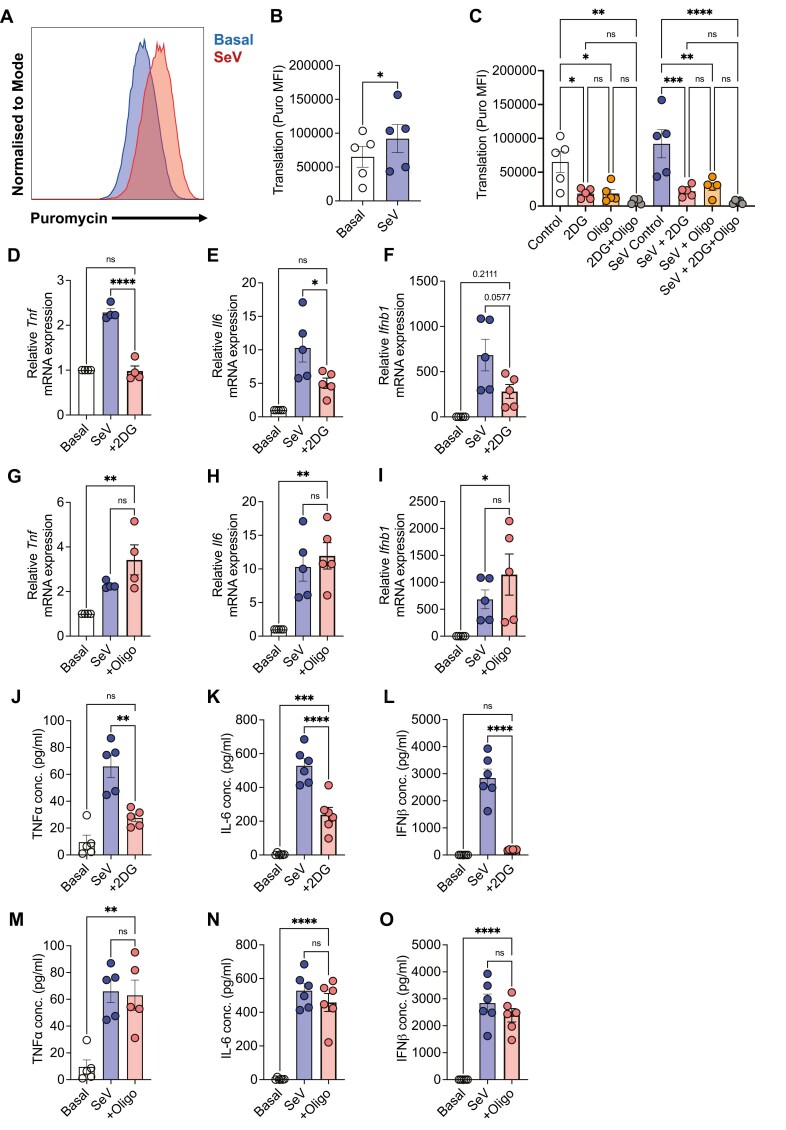
Metabolic rewiring through glycolysis is necessary for GM-DC cytokine responses. (A) Representative histogram of puromycin incorporation in basal GM-DCs and following 18-hour SeV stimulation. (B) MFI of puromycin incorporation in basal GM-DCs and following 18-hour SeV stimulation and (C) in the presence or absence of 2-DG or oligomycin or both (*n* = 5). (D) *Tnf* (*n* = 4), (E) *Il6* (*n* = 5), or (F) *Ifnb1* (*n* = 5) mRNA expression relative to *Actb* following 6-hour SeV stimulation in the presence or absence of 2-DG. (G) *Tnf* (*n* = 4), (H) *Il6* (*n* = 5), or (I) *Ifnb1* (*n* = 5) mRNA expression relative to *Actb* following 6-hour SeV stimulation in the presence or absence of oligomycin. (J) TNFα (*n* = 5), (K) IL-6 (*n* = 6), or (L) IFNβ (*n* = 6) production (pg/ml) following 18-hour SeV stimulation in the presence or absence of 2-DG. (M) TNFα (*n* = 5), (N) IL-6 (*n* = 6), or (O) IFNβ (*n* = 6) production (pg/ml) following 18-hour SeV stimulation in the presence or absence of oligomycin. Statistical analysis performed using paired student’s *t*-test or Ordinary one-way ANOVA with Tukey’s correction where appropriate.

Depending on the stimuli, DCs require different metabolic pathways to respond. While upregulation of glycolysis is required following LPS stimulation [34], the metabolic adaptations used by DCs in response to SeV infection are unknown. To understand the metabolic capacity of SeV-stimulated DCs at a single-cell level, we performed SCENITH [28]. SCENITH measures the levels of protein translation within individual cells via flow cytometry as a surrogate marker for cellular metabolic activity (e.g. energy production). The incorporation of puromycin is used to determine the rates of protein synthesis within the cell. We report a significant increase (*P* < 0.05) in puromycin incorporation (as demonstrated via geometric mean fluorescence intensity) in SeV-stimulated GM-DCs compared to untreated cells (Fig. 2A and B). Next, we determined the effect of abrogating specific metabolic pathways on protein synthesis during viral infection. The addition of 2-DG (inhibitor of glycolysis) and/or oligomycin (inhibitor of oxidative phosphorylation) to untreated and SeV-stimulated GM-DCs markedly decreased protein translation, an effect more pronounced with the simultaneous addition of both (Fig. 2C; *P* < 0.05). Furthermore, we determined if specific metabolic dependencies and capacities of GM-DCs were altered in response to SeV infection (Supplementary Fig. 1). Glucose or mitochondrial dependence refers to the reliance on glucose oxidation or OxPhos for protein synthesis, respectively. While LPS, acting as a positive control, significantly decreased the mitochondrial dependence of GM-DCs, in addition to a concomitant significant increase in the glycolytic capacity (*P* < 0.05; Supplementary Fig. 1D and F), no such metabolic switch was reported following SeV stimulation (Supplementary Fig. 1C and E). SeV-stimulated GM-DCs do not significantly change metabolic dependencies but instead display a higher degree of metabolic flexibility. No differences were observed in FAO & AAO capacity for both conditions (Supplementary Fig. 1G and H).

As cytokine production is a key function of DCs upon activation, we next examined the effect of glycolytic inhibition following SeV stimulation. GM-DCs were stimulated with SeV in the presence or absence of 2-DG and cytokine gene expression was measured. We demonstrated a significant reduction in *Tnf* (Fig. 2D; *P* < 0.0001) and *Il6* expression (Fig. 2E; *P* < 0.05), with a trending decrease in antiviral *Ifnb1* (Fig. 2F; *P* = 0.0577), following the inhibition of glycolysis. We confirmed these results at the protein level and determined that SeV stimulation following glycolytic inhibition reduced all measured cytokines (Fig. 2J–L; *P* < 0.05), with an almost complete abrogation of IFNβ (Fig. 2L; *P* < 0.0001). To further confirm the requirement of glycolysis for SeV-induced GM-DC cytokine responses, a second glycolytic inhibitor was used, Heptelidic acid (GAPDH inhibitor), and its addition also resulted in a significant reduction in cytokine production (Supplementary Fig. 2; *P* < 0.05). We also investigated the role of mitochondrial metabolism in SeV-induced cytokine responses in DCs and observed no significant differences in cytokine mRNA (Fig. 2G–2I) or protein expression (Fig. 2M–O). Hence, the inhibition of OxPhos did not affect the cytokine production by GM-DCs, emphasizing that GM-DC may have a greater dependency on glycolysis for cytokine production.

### Obesity leads to defective antiviral responses in mice

**Figure 3:**
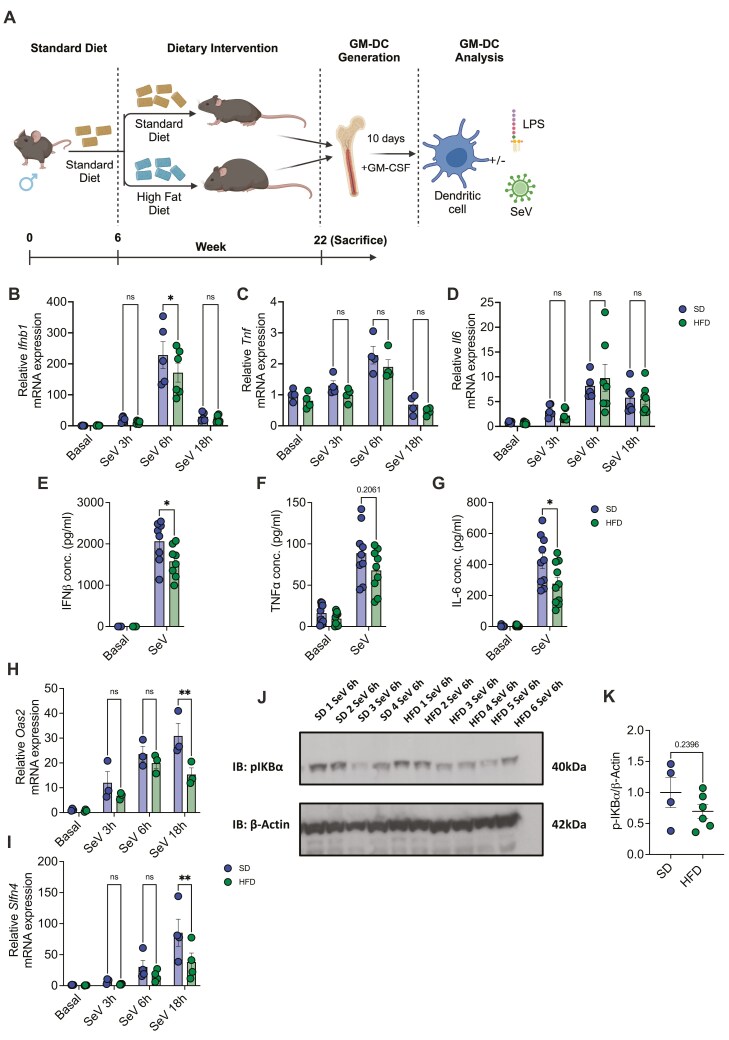
Obesity results in defective antiviral responses. (A) Schematic of the 16-week diet-induced model of obesity, where C57BL6/J mice were placed on either a standard diet (SD) or high-fat diet (HFD) for 16 weeks, after which bone marrow precursors were obtained and GM-DCs were generated (created with BioRender.com). (B) *Ifnb1* (SD *n* = 5, HFD *n* = 6), (C) *Tnf* (*n* = 4) or (D) *Il6* (SD *n* = 6, HFD *n* = 7) mRNA expression relative to *Actb* following SeV stimulation at different time points between SD and HFD. (E) IFNβ (*n* = 8), (F) TNFα (*n* = 9) or (G) IL-6 (*n* = 10) production (pg/ml) following 18-hour SeV stimulation. (H) *Oas2* (*n* = 3) or (I) *Slfn4* (*n* = 4) mRNA expression relative to *Actb* following SeV stimulation at different time points between SD and HFD. (J) Immunoblot of phospho-IKBα (40kDa) and β-Actin (42kDa) expression following 6-hour SeV stimulation of SD and HFD GM-DCs. (K) Densitometry of p-IκBα expression relative to β-Actin (SD *n* = 4, HFD *n* = 6). Statistical analysis performed using unpaired student’s *t*-test or Two-way ANOVA with Tukey’s correction where appropriate.

Given that PWO demonstrate increased susceptibility to viral infection, and the increased prevalence of obesity worldwide, we next aimed to examine if obesity impacts the antiviral response in DCs. We utilized a diet-induced model of obesity to determine the impact of adiposity on antiviral immune responses. A schematic overview of this 16-week HFD model is depicted in Fig. 3A. Supplementary Fig. 3A and B confirms that HFD mice had greater average weight and higher average weight gain each week compared to SD mice (*P* < 0.001). Furthermore, after 16 weeks, on the day of sacrifice, the HFD mice weighed significantly more than the SD mice (Supplementary Fig. 3C; *P* < 0.0001), and the HFD mice had approximately double the weight of adipose tissue compared to SD mice (Supplementary Fig. 3D; *P* < 0.0001).

Following both SD and HFD diets, GM-DCs were generated *in vitro,* stimulated with SeV, and cytokine expression was determined. Peak cytokine gene expression was observed at 6 hours (Fig. 3B–D). While a non-significant trending decrease in *Tnf* was observed in SeV-stimulated HFD GM-DCs compared to SD GM-DCs (Fig. 3C), no differences were observed between SD and HFD GM-DC *Il6* expression (Fig. 3D). However, the expression of antiviral *Ifnb1* was significantly reduced in HFD GM-DCs at 6 hours post-SeV infection (Fig. 3B; *P* < 0.05). Furthermore, at the protein level, we observed a significant decrease in IL-6 and IFNβ following SeV stimulation of HFD GM-DCs compared to SD (Fig. 3E and G; *P* < 0.05).

We hypothesized that a reduction in IFNβ in HFD GM-DCs could potentially have effects on downstream interferon-stimulated genes (ISGs). IFN-induced *Oas2* yields a protein that activates ribonuclease L to inhibit viral replication via the degradation of viral and cellular RNA [35, 36], we had observed an increase in *Oas2* following SeV stimulation in our RNAseq data (Fig. 1B). Schlafens (SLFNs) are an understudied protein family activated by type I IFNs and members of this family have roles ranging from cell differentiation, proliferation, and inhibition of viral replication [37–40]. Following 18 hours of SeV infection, we demonstrate a significant decrease in the expression of these IFN-activated genes, *Oas2* and *Slfn4*, in HFD GM-DCs compared to SD (Fig. 3H and I; *P* < 0.01).

The upregulation of cytokines following viral stimulation requires the activation of various signalling pathways, including NF-κB. Given that SeV stimulation results in NF-κB activation (Fig. 1F and G), we next assessed if obesity could impair this activation. We demonstrate that SeV stimulation in GM-DCs generated from HFD mice does not differ significantly in NF-κB activation compared to mice fed a SD (Fig. 3J and K). Therefore, the antiviral perturbations conferred by the HFD are potentially mediated via a different mechanism.

### Antiviral immune responses in GM-DC are underpinned by certain obesity-driven metabolic defects

**Figure 4:**
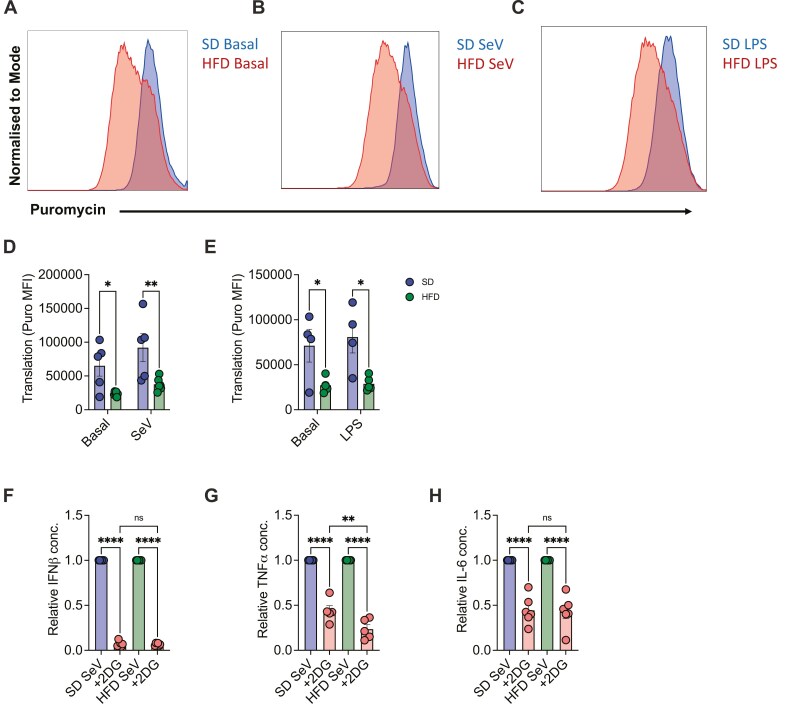
Obesity-driven defects in metabolism. Representative histograms of puromycin geometric mean fluorescence intensity (MFI) in SD and HFD GM-DCs (A) basally, (B) following 18-hour SeV stimulation or (C) following 18-hour LPS stimulation. Puromycin incorporation (MFI) in SD and HFD GM-DCs (D) following 18-hour SeV stimulation (SD *n* = 5, HFD *n* = 6) or (E) following LPS stimulation (SD *n* = 4, HFD *n* = 5). Relative (F) IFNβ (*n* = 6), (G) TNFα (*n* = 5) or (H) IL-6 (*n* = 6) concentration following 18-hour SeV stimulation in the presence or absence of 2-DG. Statistical analysis performed using Ordinary one-way ANOVA with Tukey’s correction or Two-way ANOVA with Bonferroni’s correction where appropriate.

Obesity has previously been shown to affect the metabolism of various immune cells and consequently their function. We hypothesized that the HFD could impact GM-DC metabolism, thus resulting in defective antiviral immune responses. Using SCENITH, we measured puromycin incorporation in SD and HFD GM-DCs basally and following SeV or LPS (positive control) stimulation. Firstly, basal HFD GM-DCs had a significant reduction in puromycin incorporation compared to SD GM-DCs (Fig. 4A and D; *P* < 0.05). SeV stimulation increased protein translation within HFD GM-DCs compared to basal (Supplementary Fig. 4A; *P* < 0.05), with no significant difference observed in LPS-stimulated HFD GM-DCs compared to basal (Supplementary Fig. 4B). We report a significant decrease in puromycin incorporation in HFD SeV-stimulated (Fig. 4B and D; *P* < 0.01) and LPS-stimulated (Fig. 4C and E; *P* < 0.05) GM-DCs, compared to SD GM-DCs. Furthermore, upon examination of metabolic dependencies and capacities, we demonstrate that both SD and HFD GM-DCs have comparable results following SeV infection (Supplementary Fig. 4C–J). Basal SD and HFD GM-DCs have similar glucose and mitochondrial dependence (Supplementary Fig. 4C–F). LPS stimulation compared to basal resulted in the same decrease in mitochondrial dependence (Supplementary Fig. 4F) and increase in glycolytic capacity (Supplementary Fig. 4H) in HFD GM-DCs, similar to SD (Supplementary Fig. 1D and F).

As approximately 50% of cellular energy is utilized for protein synthesis and we demonstrated the importance of glycolysis for cytokine production (Fig. 2J–L), we next investigated the impact of glycolytic inhibition on HFD GM-DC cytokine production. We normalized SD and HFD GM-DC cytokine production to determine whether 2-DG impacted both conditions to the same extent. In the presence of 2-DG, relative IFNβ (Fig. 4F) and IL-6 (Fig. 4H) production were comparable for SD and HFD GM-DCs following SeV stimulation. The same abrogation of IFNβ was observed in HFD GM-DCs (Fig. 4F). However, relative TNFα levels were reduced in HFD GM-DCs compared to SD when glycolysis was inhibited (Fig. 4G). These results demonstrate that HFD GM-DCs utilize the same metabolic pathways within the cell, including glycolysis for cytokine production; however, overall, the rates of protein synthesis, including cytokine production, are reduced within HFD GM-DCs compared to SD.

## Discussion

Obesity is a progressive, multi-factorial disease, correlated with over 200 chronic co-morbidities [4, 41]. During the H1N1 and COVID-19 pandemic, obesity was associated with increased susceptibility, morbidity, and mortality to the influenza A (subtype H1N1) and severe acute respiratory syndrome-coronavirus-2 (SARS-CoV-2) viruses, respectively [5, 6]. Vaccinations are utilized as a means of protection against these viruses. However, obesity is also linked with reduced vaccination efficacy [11–13]. Given that DCs are essential in inducing robust effector and memory T-cell responses during vaccination and indeed infection, we sought to investigate the impact of obesity on their function. In this study, we report the induction of a potent antiviral response in GM-DCs in response to the murine-specific single-stranded RNA virus, SeV. We show for the first time that this response is coupled with a boost in protein translation concomitant with an increased dependency on glycolysis. Furthermore, we demonstrate for the first time, that obesity impairs this antiviral response to SeV in GM-DCs as demonstrated by reduced induction of key pro-inflammatory and antiviral mediators. Finally, we report that this impairment in antiviral responses is associated with an impairment in protein synthesis. Collectively our data demonstrate that SeV can induce a robust antiviral response in GM-DCs which is reliant on cellular metabolism and increased protein translation. Furthermore, we demonstrate that obesity impairs this response.

The transcriptional programming of GM-DCs following SeV stimulation has not been previously investigated. We report that SeV stimulation of GM-DCs results in the transcriptional remodelling of processes spanning from immune signalling, antigen processing to metabolic processes, with a particular emphasis on genes involved in the antiviral response. This agrees with previously published studies in which SeV stimulation has been shown to induce an antiviral response in GM-DCs characterized by increased type I IFN and pro-inflammatory cytokine secretion and increased co-stimulatory marker expression (CD80 and CD86) [42]. In order for an antiviral response to be initiated, SeV must be detected on the cell either by cytosolic pattern recognition receptors, such as RIG-I [43] and MDA-5 [29]. In agreement with this, our transcriptomic analysis identified an increase in expression in the viral sensors *Ddx58* (RIG-I) and *Ifih1* (MDA-5) following infection. Importantly, these viral genomic sensors activate several diverse pathways, culminating in increased gene expression and the development of a robust antiviral response. We confirmed that SeV infection induced NF-κB activation in GM-DCs which is in agreement with previous studies [42]. Furthermore, our transcriptomic analysis revealed that multiple genes involved in GM-DC activation and maturation such as co-stimulatory molecules (*Cd40*), cytokines (*Il6/Il27*), and chemokines (Cxcl9/Cxcl10/Cxcl11) were all upregulated in response to SeV infection. Further evidence is that DCs are capable of generating a robust antiviral response to SeV infection. Transcriptomic analysis of DCs from people infected with SARS-CoV-2 also displays alterations in the expression of co-stimulatory (*CD40/CD80/CD86*) and maturation (*CD83*) markers and MHC class II molecules [44]. Moreover, as cytokine production is a key DC effector function, we validated our RNAseq data by measuring the expression and production of various antiviral (*Ifnb1*/IFNβ) and pro-inflammatory cytokines (*Tnf*/TNFα and *Il6*/IL-6) by real-time PCR and ELISAs. Increased mRNA expression gave rise to increased protein production of these key cytokines, production of these cytokines following SeV stimulation has previously been shown [25]. Taken together, we can report that SeV induces a robust transcriptional change in GM-DCs, resulting in a potent antiviral response.

Reprogramming of DC metabolism is required for DC fate and function. Due to the rarity of conventional DCs, GM-DCs have largely been used to study DC metabolism [45, 46]. We sought to determine the metabolic requirements during viral infection with SeV, compared to the more commonly used TLR agonist, LPS. While the effect of LPS stimulation on GM-DC function and subsequent metabolism has been extensively studied, the role of SeV infection on GM-DC metabolism is entirely unknown. We report that activation of DCs by SeV causes a significant increase in protein translation, an established surrogate marker of cellular metabolism [28]. Using various inhibitors of glycolysis and oxidative phosphorylation—the two main energy pathways in the cell—we investigated the potential role these pathways may play in GM-DC response to SeV. We report that inhibition of glycolysis (confirmed with the two inhibitors, 2-DG and heptelidic acid) caused a significant decrease in cytokine mRNA expression and protein translation, demonstrating a key role of glycolysis for effective antiviral responses to SeV. Viruses can target metabolic pathways at different stages, reviewed by Girdhar *et al.* [47]. Influenza-infected DCs significantly increased glycolysis; however, pyruvate, glutamine, and free fatty acids were also shown to play a role in effective DC function during influenza A infection [48]. However, OxPhos inhibition did not significantly affect cytokine mRNA expression or indeed protein production suggesting that the initial antiviral response to SeV in GM-DCs is more reliant on glycolysis. Previous studies in DC metabolism have reported what is known as a metabolic ‘switch’ in which cells downregulate one metabolic pathway in preference for another (usually OxPhos and glycolysis, respectively) [49]. Indeed, Everts *et al*. [34] observed a decrease in OxPhos following LPS stimulation of GM-DCs. We suggest that while LPS stimulation of DCs results in a metabolic switch from OxPhos to glycolysis, this may not be representative of what occurs following a viral infection such as SeV, a similar observation was demonstrated in influenza A-infected DCs [48]. In support of this, we demonstrate that SeV- or LPS-stimulated GM-DCs exhibited contrasting metabolic dependencies and capacities. LPS-stimulated GM-DCs had reduced mitochondrial dependence and increased glycolytic capacity compared to untreated GM-DCs, as opposed to SeV-stimulated GM-DCs, where no differences were observed. Conventional DC subsets, derived from Flt3L-cultured DCs (FL-DCs), treated with LPS also displayed a decrease in mitochondrial dependency and an increase in glycolytic capacity [28], correlating with our current observation in GM-DCs, likely due to the long-term commitment towards glycolysis following LPS treatment of GM-DCs. Further work is required to understand the complexities of SeV metabolic dependencies and capacities which appear unique to those observed following bacterial ligand infection. While our study is the first to examine the immunometabolic response of DCs to SeV, previous studies have aimed to explore what metabolic adaptations are induced in DCs following viral infection. Specifically, Rezinciuc *et al.* [48] compared influenza A virus stimulation to other TLR ligands and demonstrated differences in GM-DCs metabolic responses, where the glycolytic switch was not observed, influenza A-infected DCs still significantly increased glycolysis but the decrease in oxygen consumption rate (OCR) did not occur, unlike with other TLR ligands (R848/LPS) where the expected OCR decrease was observed. Following the discovery that SeV-mediated immune responses are mediated via glycolysis, we next aimed to determine how these metabolic pathways could be impacted by obesity.

We previously observed decreased circulating DCs in PWO, along with defective cytokine production and decreased maturation marker expression following LPS stimulation [19]. Based on this observation and our knowledge of obesity-induced alterations in the metabolism of other immune cells [10, 22], we hypothesized that obesity may result in alterations in DC metabolism which in turn, affects DC function. We first investigated if obesity altered GM-DC response to SeV using a 16-week diet-induced model of obesity. SeV was used as a stimulant *in vitro*, the effects of SeV *in vivo* on DCs within the periphery is an avenue worth exploring to determine how obesity affects the viral and metabolic responses of conventional DCs (cDCs), especially within metabolic tissues. Notably, we observed a significant decrease in cytokine production, IL-6, and IFNβ, key cytokines required for T-cell polarization and antiviral responses, respectively, in GM-DCs derived from mice fed a HFD. Obesity has been shown to impact murine dendritic cells (DCs) differently based on their localization. A study by van der Zande *et al.* [50] recently demonstrated that obesity promoted DC activation and maturation in murine adipose tissue and liver which correlated with perturbations in downstream T helper cell populations, for example, an observed increase in Th1 cells in adipose tissue and liver. Chen *et al.* [21] also found an increase in splenic DCs of HFD mice but these DCs displayed defective antigen presentation capacity. Cha *et al.* [51] demonstrated reduced *Il12, Cd40*, and *Cd83* expression in HFD GM-DCs compared to controls. Our study further shows that obesity can result in further dysfunction in DCs, especially during a viral infection, and hints at dysfunction upstream. The impact of obesity on the bone marrow compartment and haematopoiesis warrants further research.

Importantly, HFD GM-DCs have significantly decreased protein translation compared to SD GM-DCs in untreated, LPS- and SeV-stimulated conditions. Approximately half of the generated cellular energy is directed towards protein synthesis; hence further work is required to determine whether the effect of obesity we observed is caused by an energy deficit or dysfunctional protein synthesis. There were no differences in the metabolic dependencies and capacities of SD and HFD GM-DCs. We also normalized cytokine levels to their respective sample, in the presence of 2-DG, and there were largely no differences between SD and HFD GM-DCs (except TNFα). Altogether, the HFD GM-DCs use the same pathways for SeV and LPS responses and glycolysis inhibition affected cytokine production to the same extent. TNFα levels were quite low for both SD and HFD and glycolysis inhibition yielded even lower levels, which may have caused varied measurements for the two groups. Additionally, we demonstrate these changes in protein production are not due to PRR signalling, a trending decrease in NF-κB signalling is detected in HFD GM-DCs but did not yield statistical significance. A decrease in NF-κB transcription levels from poly(I:C)-activated PBMCs of PWO has been observed, and these same cells displayed decreased IFNα, IFNβ, and IL-6 production, compared to controls [52]. Whether other signalling pathways or the rates of metabolic processes within GM-DCs in HFD mice are dysfunctional warrants further investigation.

HFD splenic DCs had an impaired ability to induce IFNγ production by CD8^+^ T cells [53], and recently, HFD splenic DCs were also shown to have a reduced antigen stimulatory capacity [21]. Taken together, including the data presented in this study, it is clear that DIO may cause a dysfunctional phenotype in murine DCs. GM-DCs are ontogenically different from cDCs and are cultured *in vitro*, hence GM-DCs do not reflect potential effects of obesity on cDCs in physiological conditions. However, we observe that DCs generated from bone marrow precursors of HFD mice retain their dysfunctional phenotype after 10 days of *in vitro* culture, demonstrated via reduced rates of protein synthesis, including diminished levels of antiviral and pro-inflammatory cytokines. The potential epigenetic wiring resulting in defective DC function serves as a potential mechanism of how obesity increases host susceptibility to infection.

## Supplementary Material

kyaf001_suppl_Supplementary_Material

## Data Availability

Data from this study are available from the corresponding author upon request.
