# Supplementary material for: Obesity drives dysregulation in DC responses to viral infection
Source: Discov Immunol. 2025 Feb 6;4(1):kyaf001. doi: 10.1093/discim/kyaf001 (PMC11892430; doi:10.1093/discim/kyaf001)
Supplement: kyaf001_suppl_Supplementary_Material [file kyaf001_suppl_supplementary_material.zip › supplement captions.docx]

**Supplementary Table 1: Total DEGs between basal GM-DCs and following 18-hour SeV stimulation**

Table outlining the total number of genes differentially expressed following an 18-hour SeV stimulation compared to basal.

**Supplementary Figure 1: Metabolic dependencies and capacities of SeV- and LPS-stimulated GM-DCs**

Glucose dependence of GM-DCs following (A) 18-hour SeV (n=5) or (B) 18-hour LPS stimulation (n=4). Mitochondrial dependence of GM-DCs following (C) 18-hour SeV (n=5) or (D) 18-hour LPS stimulation (n=4). Glycolytic capacity of GM-DCs following (E) 18-hour SeV (n=5) or (F) 18-hour LPS stimulation (n=4). Fatty acid oxidation (FAO) and amino acid oxidation (AAO) capacity of GM-DCs following (G) 18-hour SeV (n=5) or (H) 18-hour LPS stimulation (n=4). Statistical analysis performed using paired student’s t-test or Wilcoxon test where appropriate.

**Supplementary Figure 2: SeV anti-viral cytokine responses require glycolysis, confirmed via a second glycolytic inhibitor**

(A) TNFα (n=4), (B) IL-6 (n=5) or (C) IFNβ (n=6) production (pg/ml) following 18-hour SeV stimulation in the presence or absence of Heptelidic acid. Statistical analysis performed using Ordinary one-way ANOVA with Tukey’s correction.

**Supplementary Figure 3: HFD induced increase in mice weight and adiposity**

(A) Average weight of mice fed on either standard diet (SD) or high fat diet (HFD), weighed weekly for 16 weeks (n=5). (B) Average weight gain of mice on SD or HFD relative to initial average week 0 weight (n=5). (C) SD and HFD mice weights on the day of the sacrifice at the end of the 16-week diet (SD n=21, HFD n=23). (D) Weights of epididymal adipose tissue extracted from SD and HFD mice on the day of sacrifice (n=18). Statistical analysis performed using area under curve (AUC) for (A), linear regression for (B) and unpaired student’s t- test for (C) & (D).

**Supplementary Figure 4: Effect of obesity on basal and stimulated GM-DC puromycin incorporation and metabolic dependencies and capacities**

Puromycin incorporation (MFI) in HFD GM-DCs (A) following 18-hour SeV stimulation (n=6) or (B) following 18-hour LPS stimulation (n=5). Glucose dependence of SD and HFD GM-DCs following (C) 18-hour SeV stimulation (n=5) or (D) 18-hour LPS stimulation (SD n=4, HFD n=5). Mitochondrial dependence of SD and HFD GM-DCs following (E) 18-hour SeV stimulation (n=5) or (F) 18-hour LPS stimulation (SD n=4, HFD n=5). Glycolytic capacity of SD and HFD GM-DCs following (G) 18-hour SeV stimulation (n=5) or (H) 18-hour LPS stimulation (SD n=4, HFD n=5). Fatty acid oxidation (FAO) & amino acid oxidation (AAO) capacity of SD and HFD GM-DCs following (I) 18-hour SeV stimulation (n=5) or (J) 18-hour LPS stimulation (SD n=4, HFD n=5). Statistical analysis performed using paired student’s t-test or Two-way ANOVA with Tukey’s correction where appropriate.
