# Supplementary material for: Obesity drives dysregulation in DC responses to viral infection
Source: Discov Immunol. 2025 Feb 6;4(1):kyaf001. doi: 10.1093/discim/kyaf001 (PMC11892430; doi:10.1093/discim/kyaf001)
Supplement: kyaf001_suppl_Supplementary_Material [file kyaf001_suppl_supplementary_material.zip › SeV Paper Revision - Supplementary Updated.pdf]

Supplementary Table 1: Total DEGs between basal GM-DCs and following 18-hour SeV stimulation

	Basal vs SeV stim.
Total differentially expressed genes	2821
Increasing	1293
Decreasing	1528

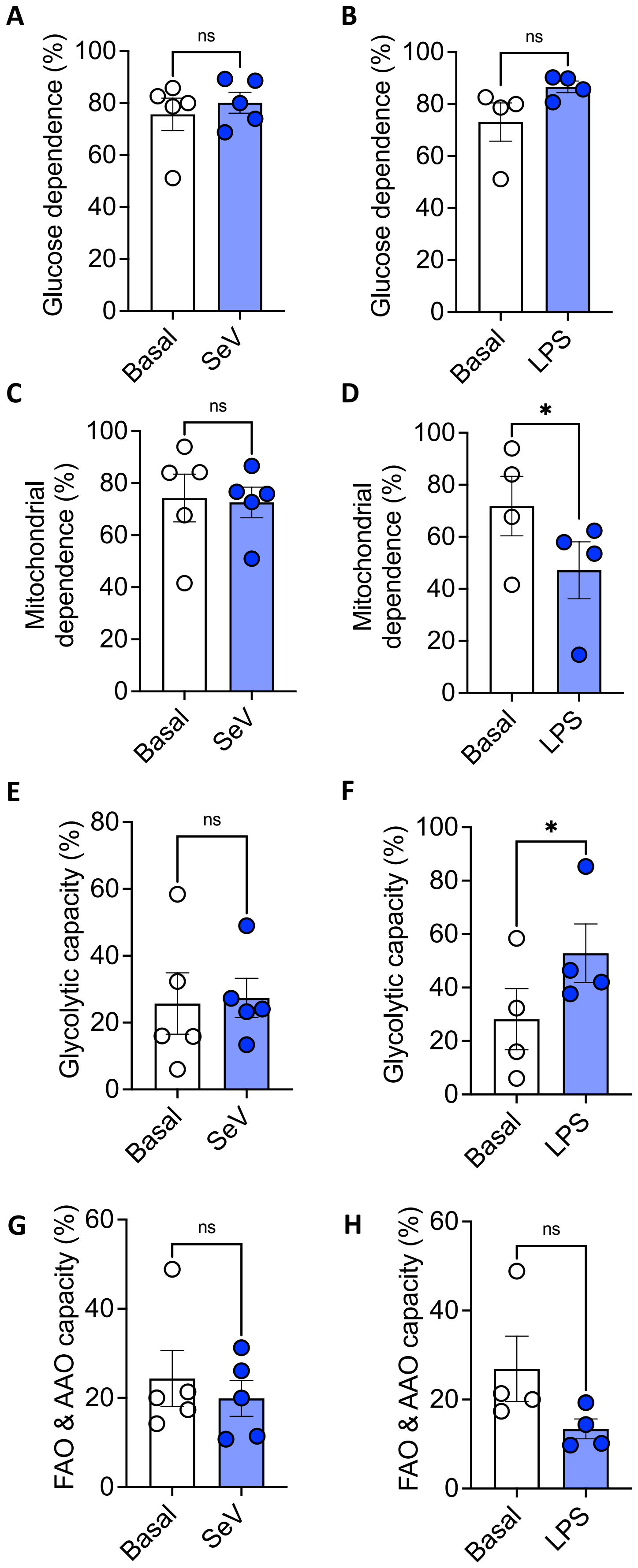

Supplementary Figure 1: Metabolic dependencies and capacities of SeV- and LPS-stimulated GM-DCs

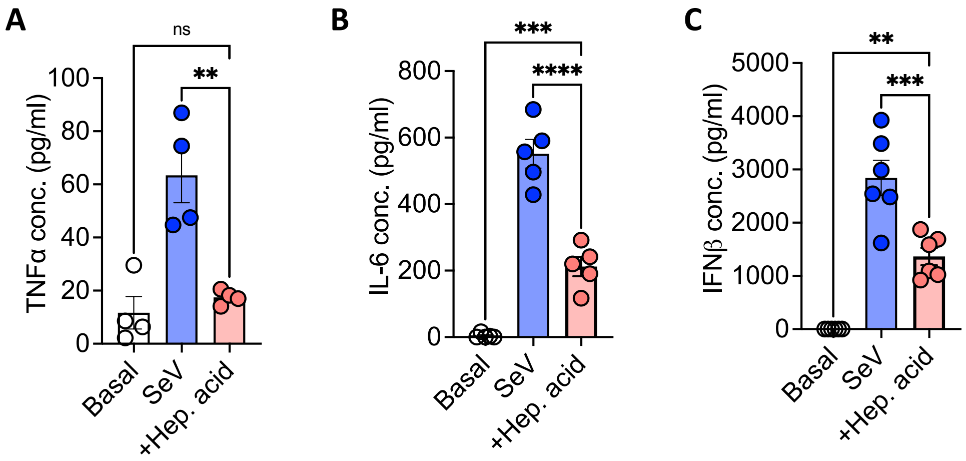

Supplementary Figure 2: SeV anti-viral cytokine responses require glycolysis, confirmed via a second glycolytic inhibitor

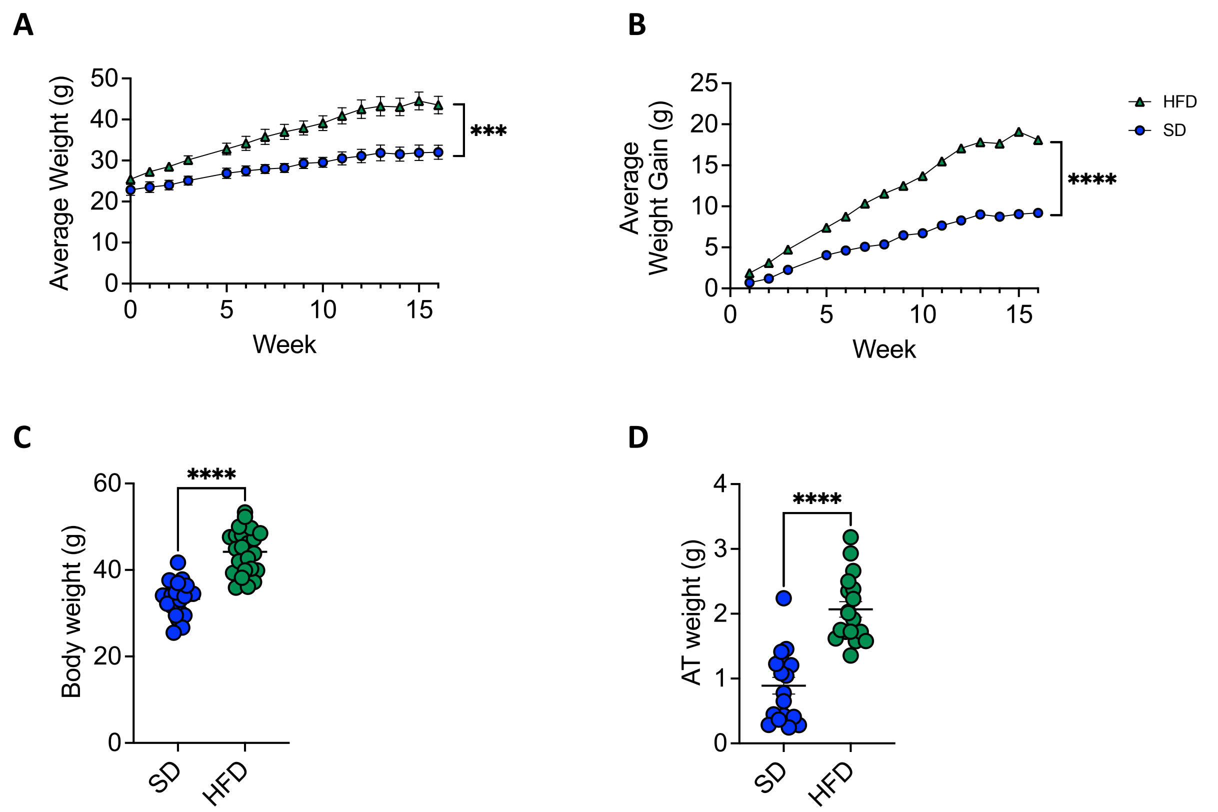

Supplementary Figure 3: HFD induced increase in mice weight and adiposity

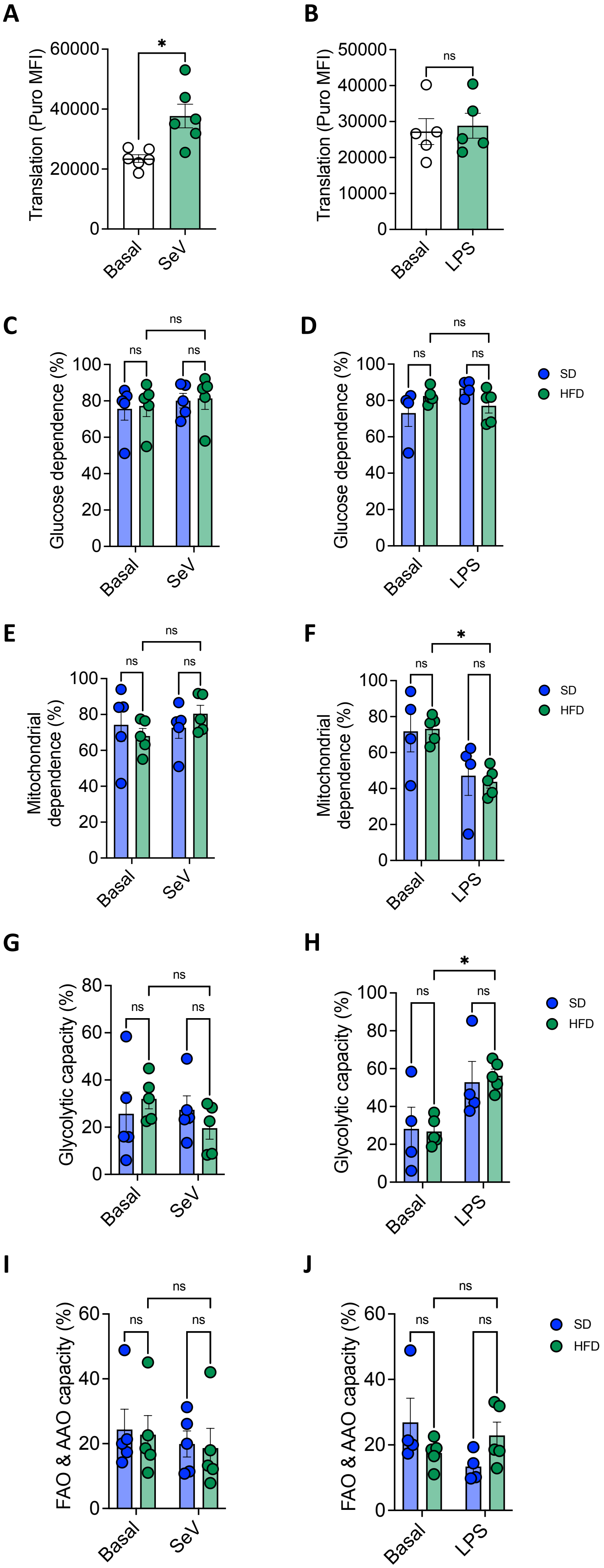

Supplementary Figure 4: Effect of obesity on basal and stimulated GM-DC puromycin incorporation and metabolic dependencies and capacities
